# Supplementary material for: Semaglutide Improves Lipid Subfraction Profiles in Type 2 Diabetes: Insights from a One-Year Follow-Up Study
Source: Int J Mol Sci. 2025 Jun 20;26(13):5951. doi: 10.3390/ijms26135951 (PMC12250273; doi:10.3390/ijms26135951)

Supplementary Table S1. Summary of the changes in lipoprotein subfractions after semaglutide treatment using a multivariate linear regression model.

Variables	Coefficient	95% CI		p-value
**Changes in large LDL subfractions (mmol/l)**				
Changes in BMI (kg/m^2)	0,010	-0,043	0,064	0,680
Age (years)	-0,009	-0,020	0,003	0,121
Sex (M/F)	-0,063	-0,350	0,224	0,642
Changes in HbA1c (%)	0,014	-0,085	0,113	0,764
**Changes in small LDL subfractions (mmol/l)**				
Changes in BMI (kg/m^2)	-0,025	-0,087	0,036	0,390
Age (years)	-0,001	-0,014	0,013	0,927
Sex (M/F)	-0,171	-0,501	0,160	0,285
Changes in HbA1c (%)	0,012	-0,103	0,126	0,828
Changes in mean LDL sizes (nm)				
Changes in BMI (kg/m^2)	0,234	-0,559	1,028	0,534
Age (years)	0,016	-0,155	0,187	0,842
Sex (M/F)	0,938	-3,294	5,170	0,640
Changes in HbA1c (%)	-0,302	-1,767	1,163	0,664
**Changes in large HDL subfractions (mmol/l)**				
Changes in BMI (kg/m^2)	0,009	-0,009	0,026	0,310
Age (years)	0,001	-0,003	0,004	0,705
Sex (M/F)	-0,090	-0,190	0,011	0,075
Changes in HbA1c (%)	0,019	-0,013	0,051	0,221
**Changes in intermediate HDL subfractions (mmol/l)**				
Changes in BMI (kg/m^2)	0,010	-0,043	0,064	0,680
Age (years)	-0,009	-0,020	0,003	0,121
Sex (M/F)	-0,063	-0,350	0,224	0,642
Changes in HbA1c (%)	0,014	-0,085	0,113	0,764
**Changes in small LDL subfractions (mmol/l)**				
Changes in BMI (kg/m^2)	0,009	-0,009	0,026	0,310
Age (years)	0,001	-0,003	0,004	0,705
Sex (M/F)	-0,090	-0,190	0,011	0,075
Changes in HbA1c (%)	0,019	-0,013	0,051	0,221

Supplementary Figure S1: Visual depiction of the electrophoresis images of the low-density lipoprotein (LDL) subfractions to illustrate the analytical process. **(a)** Representative scan image from eleven type 2 diabetic patients (Pt_{1-11}). Liposure Serum Lipoprotein Control (QC) used as an internal standard in each electrophoresis chamber. Up to seven LDL subfractions can be identified between the bands of very low-density lipoprotein (VLDL) (starting reference point) and high-density lipoprotein (HDL) (ending reference point). MidC-A represent intermediate density lipoprotein (IDL) subfractions. **(b)** Representative densitogram of a type 2 diabetic patient.

(a)

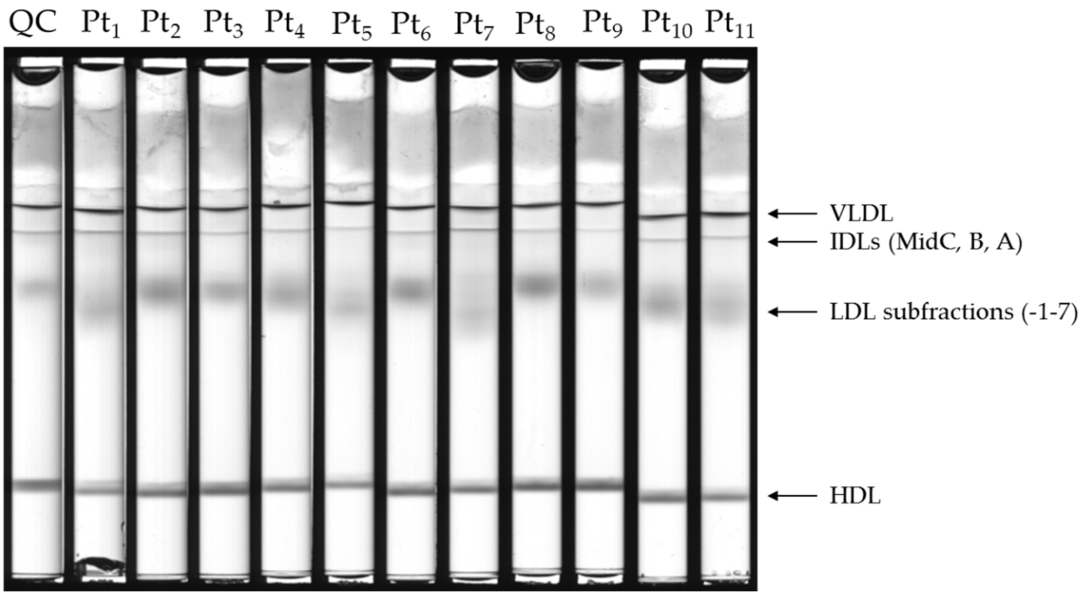

(b)

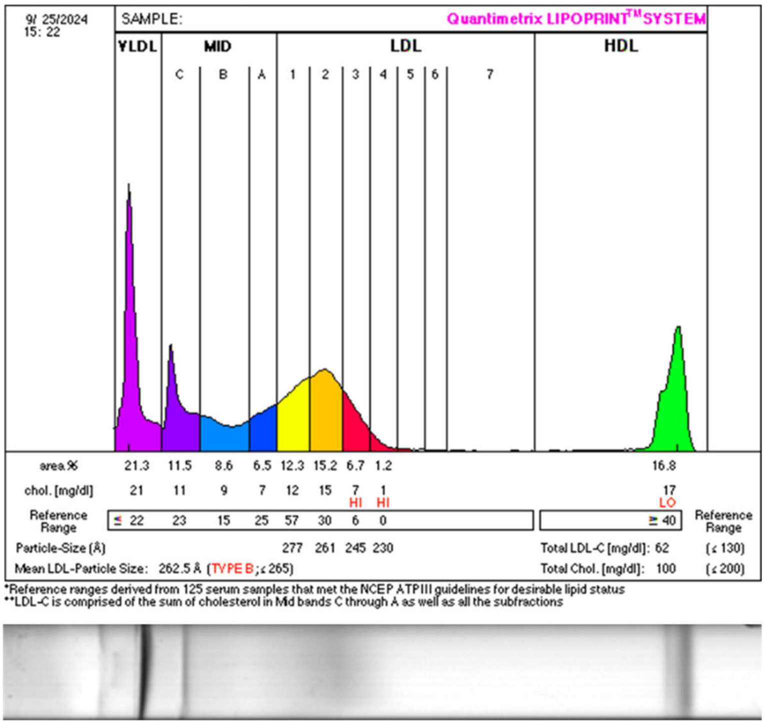

Supplementary Figure S2: Visual depiction of the electrophoresis images of the high-density lipoprotein (HDL) subfractions to illustrate the analytical process. **(a)** Representative scan image from eleven type 2 diabetic patients (Pt1-11). Liposure Serum Lipoprotein Control (QC) used as an internal standard in each electrophoresis chamber. Ten HDL subfractions can be identified between the bands of very low-density lipoprotein+low-density lipoprotein (VLDL+LDL) (starting reference point) and albumin (ending reference point). **(b)** Representative densitogram of a type 2 diabetic patient.

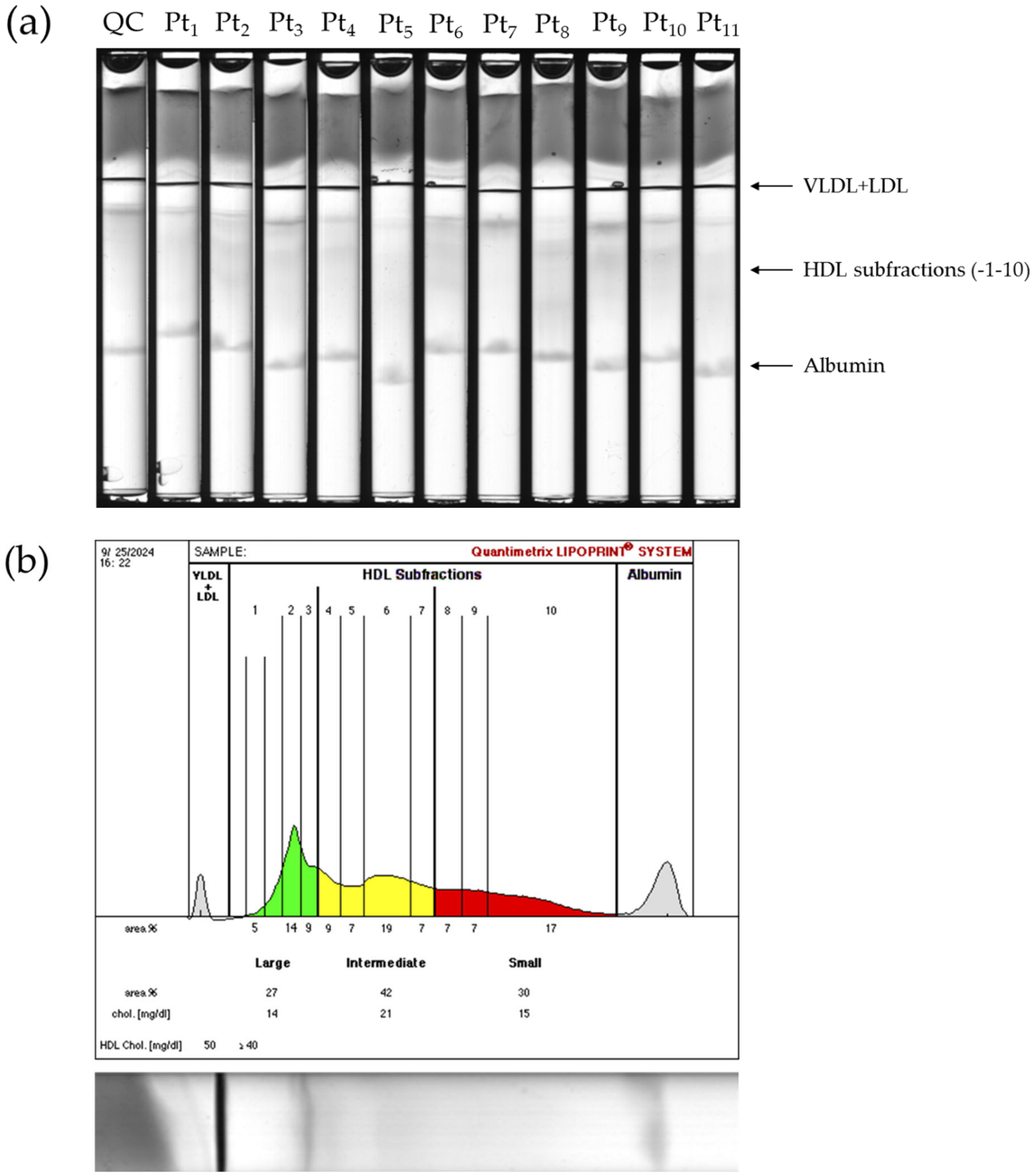

Supplement: Supplementary file 1 [file ijms-26-05951-s001.zip › ijms-3680168-supplementary.pdf]
